# Multivariate Statistical Analysis on a SEM/EDS Phase Map of Rare Earth Minerals

**DOI:** 10.1155/2020/2134516

**Published:** 2020-01-04

**Authors:** Chaoyi Teng, Raynald Gauvin

**Affiliations:** Department of Mining and Materials Engineering, McGill University, Montreal, Quebec, Canada H3A 0C5

## Abstract

The scanning electron microscope/X-ray energy dispersive spectrometer (SEM/EDS) system is widely applied to rare earth minerals (REMs) to qualitatively describe their mineralogy and quantitatively determine their composition. The performance of multivariate statistical analysis on the EDS raw dataset can enhance the efficiency and the accuracy of phase identification. In this work, the principal component analysis (PCA) and the blind source separation (BSS) algorithms were performed on an EDS map of a REM sample, assisting to achieve an efficient phase map analysis. The PCA significantly denoised the phase map and was used as a preprocessing step for the following BSS. The BSS separated the mixed EDS signals into a set of physically interpretable components, bringing convenience to the phase separation and identification. Through the comparison between the independent component analysis (ICA) and the nonnegative matrix factorization (NMF) algorithms, the NMF was confirmed to be more suitable for the EDS mapping analysis.

## 1. Introduction

Industrial demands for rare earth elements (REEs) keep rising as a result of their increasing application in high-technology electronic devices. As sources of these elements, the rare earth minerals (REMs) and their bearing ores are required to be characterized with more efficient techniques to weigh the REE concentrations. The most popular characterization system is the scanning electron microscope coupled with the X-ray energy dispersive spectrometer (SEM/EDS) due to its fast and easy operation. The annular silicon drift detector (aSDD), which enhances the count rate and spatial resolution of phase mapping, can achieve a more efficient EDS analysis [1]. However, the small proportion of REMs in the ores and the low concentration of REEs in the REMs may cause the problem of insufficient X-ray intensities for distinct peaks in the sum spectrum, resulting in omissions of rare earth phases in an EDS mapping analysis. To settle this issue, the simplest way is to extend the acquisition time to overwhelm the spectrum background, but the trade-off is the analytical efficiency.

This study investigates an alternative method, which applies the multivariate statistical analysis (MSA) on a map dataset to extract REE intensities and thus effectively shortens the data acquisition time. The MSA treats an EDS map as a set of spectral images (SIs) which encompass the spectrum at each pixel to reduce the dataset redundancy and then distinguishes the spectra for similarities and differences without predetermining elements [2]. Through combining similar SIs, the original dataset is decomposed into a limited number of components to capture the major information. In this way, the phases with different chemical constitutions can be distinguished without any biased expectation, providing preliminary knowledge for further quantitative analyses [3, 4].

The principal component analysis (PCA) is the most widely used algorithm among various MSA methods, which creates a set of orthogonal variables and orders them as decreasing variations [5–7]. Its most popular applications for X-ray microanalysis are dimensionality reduction and noise subtraction through maintaining the leading components with higher variances and discarding the rest as noise. It has been applied to EDS spectra to remove background counts and extract characteristic X-ray intensities, which shortened the necessary acquisition time [8]. It was also used to directly distinguish particles with different chemical compositions or morphologies in an EDS map [9]. However, the orthogonal constraint followed by the PCA neglect any physical consideration, which may cause difficulties in interpreting the decomposed components.

As an alternative to avoid this limitation, the blind source separation (BSS) unmixes the original dataset into a set of statistically independent components to directly capture the essential data structure [10]. Among the various BSS algorithms, the most common two applied to X-ray microanalysis are the independent component analysis (ICA) and the nonnegative matrix factorization (NMF), which both consider each decomposed component as a typical event occurring between the electron beam and the specimen [11–13]. The main difference between the two algorithms is that the NMF only allows additive combinations and prevents subtractions to force all components to be nonnegative, but the ICA allows both combinations and subtractions [14, 15]. Even though the BSS can be performed independently, it usually requires the PCA as a preliminary step to make the original dataset less correlated and decide the ummixed dimension [14]. There are more and more studies using the combination of PCA and BSS to distinguish different phases instead of the traditional elemental identification method [16, 17].

In this study, the PCA and BSS algorithms were performed on an EDS map dataset acquired on a REM sample for only 5 minutes, aimed at achieving an efficient phase analysis and finding the optimal data processing procedure.

## 2. Materials and Experimental Methods

### 2.1. Sample

The ore powders from the Nechalacho deposit (Thor Lake in Northwest Territories, Canada) were sprinkled at the bottom of a cylindrical holder, dispersed in white LR White Resin (London Resin Company Ltd., Reading, England) and then mixed thoroughly. The holder containing the dispersed powder was cured in a vacuum oven for 48 hours at a constant temperature of 60°C. After curing was complete, the sample was polished with silicon carbide papers, followed by diamond suspensions of 1 *μ*m grain size (Buehler, Lake Bluff, IL, USA) and alumina suspension of 50 nm grain size (Buehler). As the sample is a poor conductor, a thin (nominally 10-20 nm thick) coating of an amorphous carbon layer was applied using an Edwards vacuum carbon coater E306 (Edwards, Crawley, England) in order to avoid surface charging.

### 2.2. SEM/EDS Characterization

Characterizations were performed with a Hitachi SU8230 cold field emission SEM (Hitachi High-Technologies, Rexdale, ON, Canada) equipped with a Bruker Flat Quad XFlaxh® 5060F aSDD (Bruker Nano, Berlin, Germany) with a 60 mm^2^ collection area. Two sets of EDS maps were acquired for 5 minutes and 60 minutes in the same area at the condition listed in Table 1 using the Bruker ESPRIT software (version 1.9) (Bruker Nano). The probe current was measured with a Faraday cup mounted on the column with a NanoPico picoammeter from Hitachi (Hitachi High-Technologies), and the total counts were recorded by the Bruker ESPRIT.

### 2.3. Phase Map Analysis

**Figure 1:**
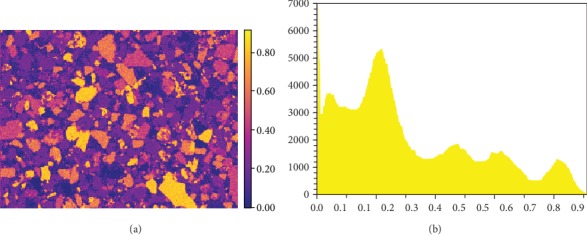
Si-K*αf*-ratio map (a) and its histogram (b).

To obtain the phase map, the conventional EDS qualitative maps were quantified by Bruker ESPRIT software to acquire the net intensity maps, and then the *f*-ratio of each element at every pixel was calculated using

(1) $$\begin{array}{c} f_{i}=\frac{I_{i}}{\sum_{i}^{N}I_{i}}, \end{array}$$

where *I*_*i*_ is the net X-ray intensity of element *i* in an N-element system from one spectrum [18]. Using Equation (1), the qualitative map of each element was converted to its *f*-ratio map, and a histogram of the *f*-ratio values was generated in the meantime. The figure displays the *f*-ratio map of Si-K*α* and its histogram (Figures 1(a) and 1(b), respectively) as an example: the number of peaks in the histogram refers to the number of minerals containing Si. Then the mineral phases were manually defined with the ranges of the *f*-ratio values of the constituted elements according to the histograms, and an open source Python script, pyPhaseMap (available on GitHub as pyphasemap repository), was run to convert the elemental *f*-ratio maps into a phase map [1, 19]. The *f*-ratios do not represent the real composition, but they are proportional to the concentrations of the corresponding elements. The adjustable *f*-ratio range is important to analyze minerals as their chemical compositions are usually not constant. The area fraction of each phase was calculated to evaluate the phase maps acquired at different conditions. The major phases were identified in the major-phase maps, and the distributions of the REMs were illustrated through overlaying the REM-phase maps with the SE image.

### 2.4. Multivariate Statistical Analysis

The Python-based open source, Hyperspy [20], was used to perform the MSA, including the PCA, and the BSS using ICA and NMF algorithms, on the 5-minute map. The number of pixels in the map is 512∗384, so there are 512∗384 channels of the SI dataset. The singular value decomposition algorithm was used for the PCA, and the cumulant-based algorithm was used for the ICA. The explained variance ratio of the components decomposed by the PCA was plotted against the component index in a scree plot, which is helpful to estimate the number of components remaining for the denoising analyses and the output dimensions for the following BSS analyses, including the ICA and NMF treatments. In the BSS analyses, a new dataset was reconstructed with the selected components, and the modified X-ray intensity maps were retrieved from it. And then, the improved phase map analysis was performed with the modified X-ray maps. The 5-minute REM maps, including the original one and the MSA-modified ones, were evaluated by counting the correctly and incorrectly identified pixels, considering the 60-minute map as a reference: the pixels identified as rare earth phases both in the 5-minute map and in the 60-minute map were counted as the correct phases, but those only identified in the 5-minute map were counted as the noise.

## 3. Results and Discussion

### 3.1. The Denoising Analysis with the PCA

**Figure 2:**
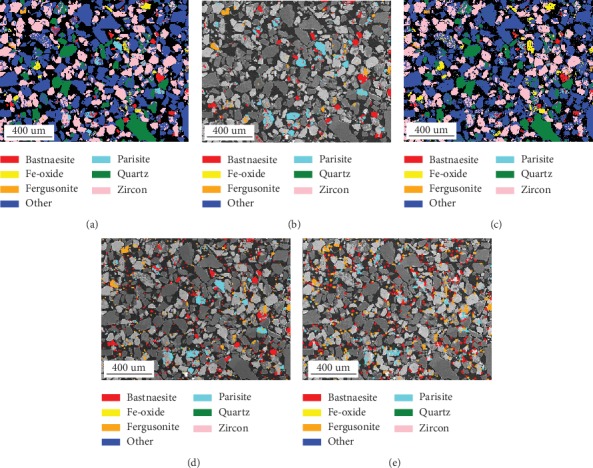
Phase maps acquired at the conditions listed in Table 1: (a) 60-minute major-phase map; (b) 60-minute REM-phase map; (c) 5-minute major-phase map; (b) 5-minute REM-phase map; (e) 5-minute noise map.

Figure 2 displays the phase maps acquired at the conditions listed in Table 1. In the phase maps, only the major phases were identified, and a large amount of the minor phases was labeled as “Other.” The 60-minute maps, including the major-phase map (Figure 2(a)) and the REM-phase map (Figure 2(b)), are much clearer compared with the 5-minute maps (the major-phase map and the REM-phase map, Figures 2(c) and 2(d), respectively) as a result of the approximate 10 times collected X-ray counts. The main rare earth carriers in this deposit: bastnaesite (Ce,La,Y)F(CO_3_), parisite (Ca(Ce,La)_2_(CO_3_)_3_F_2_), and fergusonite (YNbO_4_) [21], were identified in the maps. In the three REMs, the main REEs are La, Ce, Pr, and Nd, so these elements were used for the phase identification in this work.  Figure 2(e) is the spatial difference map which was computed by subtracting the phases in Figure 2(b) from Figure 2(d) to show the pixels misidentified as the rare earth phases in the 5-minute map. These misidentified pixels are concentrated in the mounted epoxy resin and should be removed to make the real mineral phases more visible. The comparisons shown in Figure 2 confirm that the acquisition time of 5 minutes is insufficient for an accurate phase identification.

**Figure 3:**
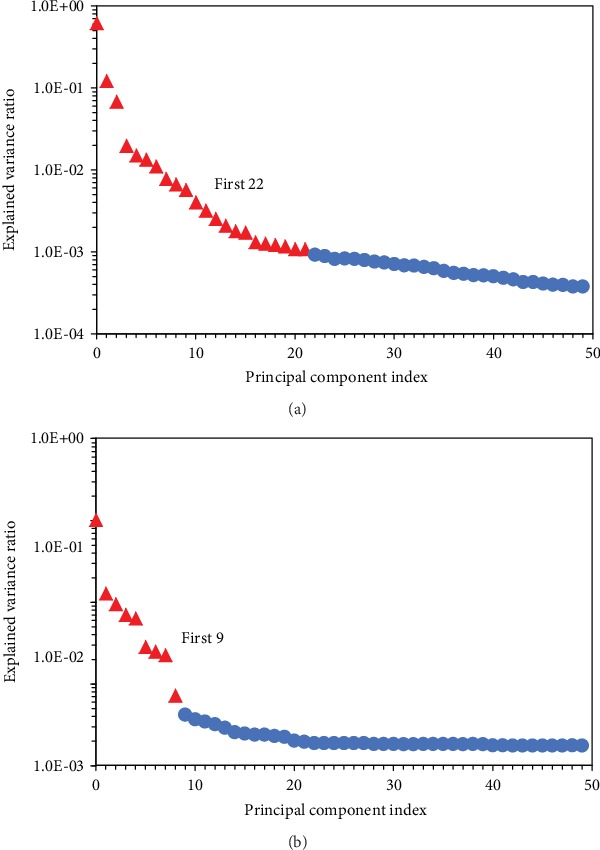
Explained variance ratio of the first 50 PCA components: (a) routine PCA and (b) weighted PCA.

In order to improve the 5-minute map, the PCA algorithm was executed to decompose the original map, and the variances of the first 50 components are plotted against their index in Figure 3. The routine PCA and the weighted PCA were both applied on the dataset, and their scree plots are displayed in Figures 3(a) and 3(b), respectively. As shown in Figure 3(a), the leading 22 components have relatively higher variances compared to the rest, but the weighted PCA algorithm revealed the significantly higher variances of the first 9 components. A compromise should be made in the component selection: all the useful information ought to be included, but the less components remaining can effectively shorten the processing time. However, the physical meaning of each component cannot be interpreted, so a direct component selection for the new dataset construction cannot be achieved.

**Figure 4:**
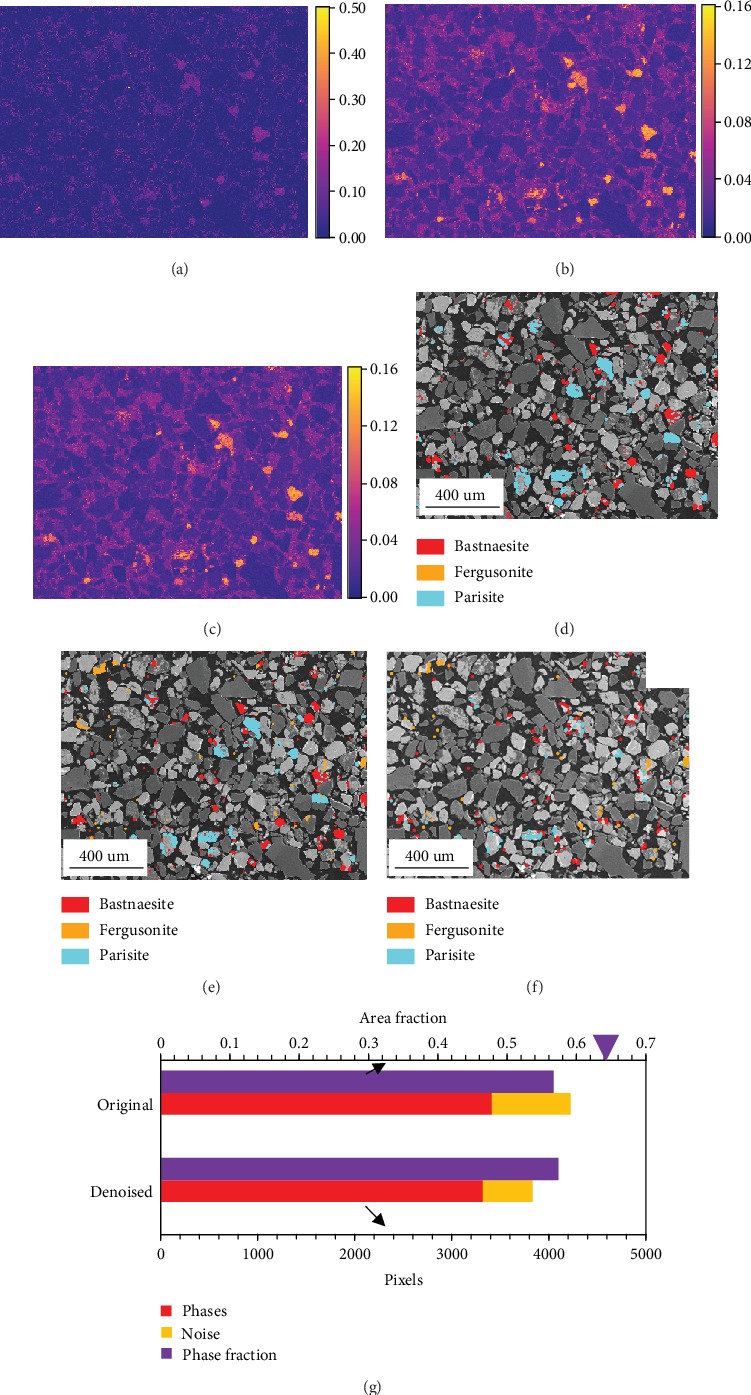
Denoising analyses with PCA on the 5-minute map. (a) Original La-L*αf*-ratio map; (b) denoised La-L*αf*-ratio map with the first 9 weighted PCA components; (c) denoised La-L*αf*-ratio map with the first 22 routine PCA components; (d) REM-phase map with the first 9 weighted PCA components; (e) REM-phase map with the first 22 routine PCA components; (f) noise map of the 22-component denoised 5-minute map; (g) bar chart of the phase identification.

The denoising analyses were performed with the first 9 and 22 components after the execution of the weighted and routine PCA algorithms, respectively, and the results are shown in Figure 4. As explained previously that the phase identification was processed with the elemental *f*-ratio maps which were computed with the elemental intensity maps by applying Equation (1) at each pixel, the *f*-ratio map processing is critical to the phase map analysis. Figures 4(a)–4(c) show the *f*-ratio maps of La-L*α* (4.65 keV) as examples: Figure 4(a) is the original La-L*αf*-ratio map, and Figures 4(b) and 4(c) are the denoised La-L*α* f-ratio maps retrieved from the first 9 and 22 components. According to Figures 4(b) and 4(c), the major La-bearing phases are among the first 9 components as the two maps do not have significant differences.

According to Equation (1), the *f*-ratio is a normalized value and is proportional to the concentrations of the corresponding elements. Since most minerals do not have constant chemical compositions, the computation of the *f*-ratio facilitates the phase identification by setting the threshold of each phase using the elemental *f*-ratio values, and the phase map is processed by applying the phase identification at each pixel. Figures 4(d) and 4(e) display the denoised REM-phase maps with the first 9 (weighted PCA) and 22 (routine PCA) components, which are both clearer than the original 5-minute map. However, when only the first 9 components were kept, the information of the orange phases (fergusonite) was totally discarded (Figure 4(d)). Thus, the threshold of 22 is more appropriate to achieve both accurate phase identification and noise reduction.

Figure 4(f) shows the spatial difference map between the denoised 5-minute map using the first 22 routine PCA components and the 60-minute map. Compared to Figure 2(e), after the PCA denoising analysis, a large amount of noise was removed from the phase map.  Figure 4(g) statistically compares the rare earth phases (red bars) and the noise (yellow bars) identified in the original 5-minute map and the 22-component denoised one. The two maps have similar pixels correctly identified as REM, but the modified map has significantly less noise. The purple bars represent the sum area fraction of all mineral phases, which show that the total identified pixels in the two maps are similar, but less than that in the 60-minute map (the purple triangle). Since the powder was mounted in the epoxy resin, the sum fraction of all mineral phases does not equal unity. Either the observation on the maps or the data summarized in the chart confirms that the decomposition of the original dataset and the reconstruction with selected PCA components can achieve the reduction of misidentified pixels and a more accurate phase identification.

Since there are a large number of minerals in this sample, the number of remaining components should be carefully checked in order to save processing time and avoid phase omissions. However, taking this 5-minute map as an example, the optimal denoising threshold might be a number between 9 and 22, but the detailed selection cannot be further made due to the unknown information in each component. In this case, the BSS is a more convenient method as each component can be physically interpreted.

### 3.2. ICA-Based and NMF-Based BSS Analyses

Two BSS algorithms, the ICA and NMF, were executed on the 5-minute dataset, and the output dimension was set to 22 according to the previous PCA results. A direct phase separation was achieved with the two algorithms: each BSS component was distinguished in the form of a multielement X-ray intensity map and its sum spectrum, which presents the phases with similar chemical compositions. The spectrum is helpful for phase identification, and the map directly shows the phase distribution.

**Figure 5:**
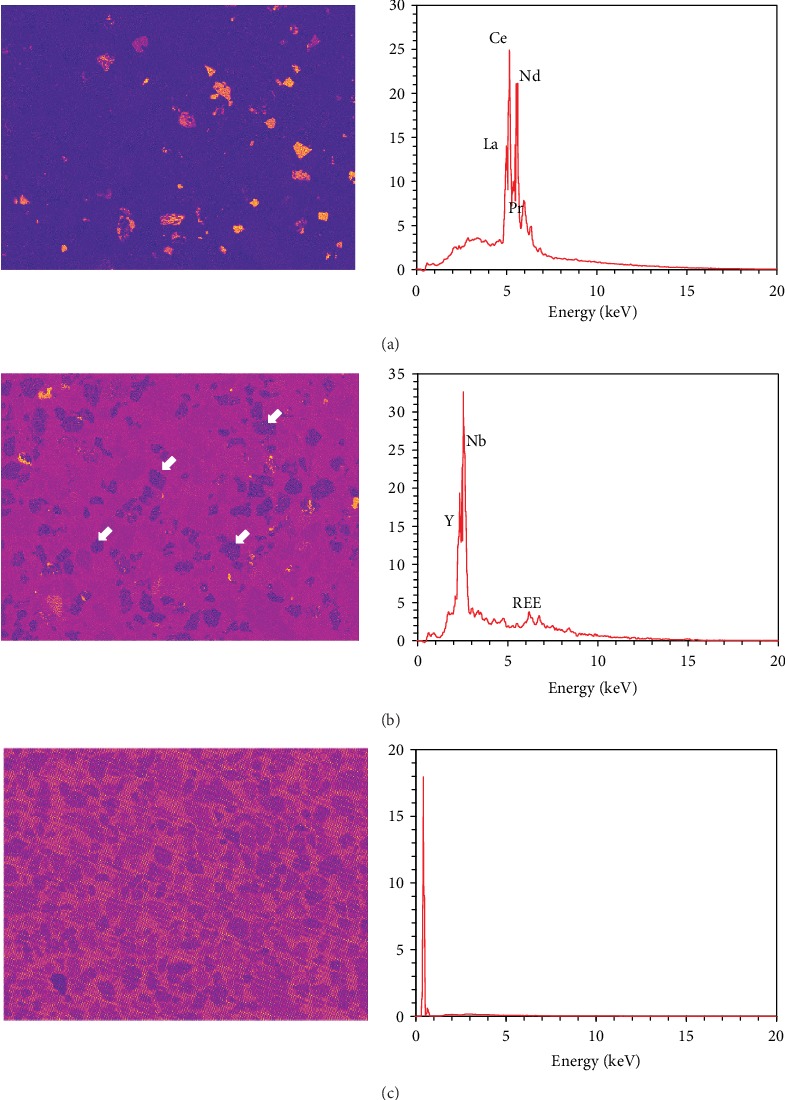
RE-components separated by ICA-based BSS: (a) bastnaesite and parisite component; (b) fergusonite component; (c) epoxy component.

Figure 5 shows the rare earth components (RE-components) separated by the ICA algorithm, including the bastnaesite and parasite (Figure 5(a)) and the fergusonite (Figure 5(b)). Remarkably, there are some non-rare earth phases presenting darker particle profiles compared with the epoxy background in the distribution map (as arrowed in white in Figure 5(b)). Since the ICA algorithm allows both combinations and subtractions, the subtraction calculation may result in negative values and physically uninterpretable results in an EDS map. Except for the mineral phases, the component of epoxy resin was also distinguished from the original dataset, as shown in Figure 5(c).

The ICA makes the component selection much easier, but the recognition power for the phase separation is decided by the preset output dimension. As shown in Figure 5(a), the bastnaesite and the parisite were considered one component when the dimension was set to 22. If further differentiation between the two phases needs to be processed, there will be repetitive attempts to set the output dimension and perform the data separation as the components are unpredictable. In addition, the computation time is also a considerable issue, which limits the output dimension not to set to a large number. Thus, it is preferred to reconstruct a new dataset with selected components, retrieve the modified X-ray intensity maps, and then perform a phase map analysis with the manual identification.

**Figure 6:**
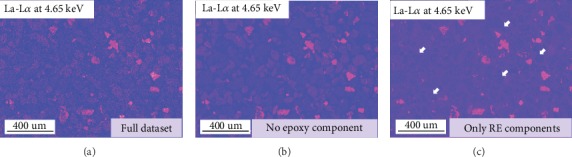
La-L*α* intensity maps retrieved from ICA datasets.

Regarding the retrieval of the modified X-ray intensity maps, the different component selection for the new dataset reconstruction may lead to different results.  Figure 6(a) shows the La-L*α* intensity map retrieved from the full dataset without any component exclusion. The non-rare earth phases can be obviously observed as the collected X-ray counts are insufficient. Also, the noise collected from the epoxy background is also shown in this map. As shown in Figure 5(c), the epoxy background component was also identified and separated from the original dataset, so this component could be excluded when the new dataset is reconstructed. The modified La-L*α* intensity map retrieved from the dataset excluding the epoxy resin component is shown in Figure 6(b). This map is slightly clearer than Figure 6(a), but other mineral phases are still visible.  Figure 6(c) shows the map retrieved only from the RE-components, which thoroughly avoids the interferences from other phase signals. However, the uninterpretable dark particle profiles are also observed in Figure 6(c) (as arrowed in white).

**Figure 7:**
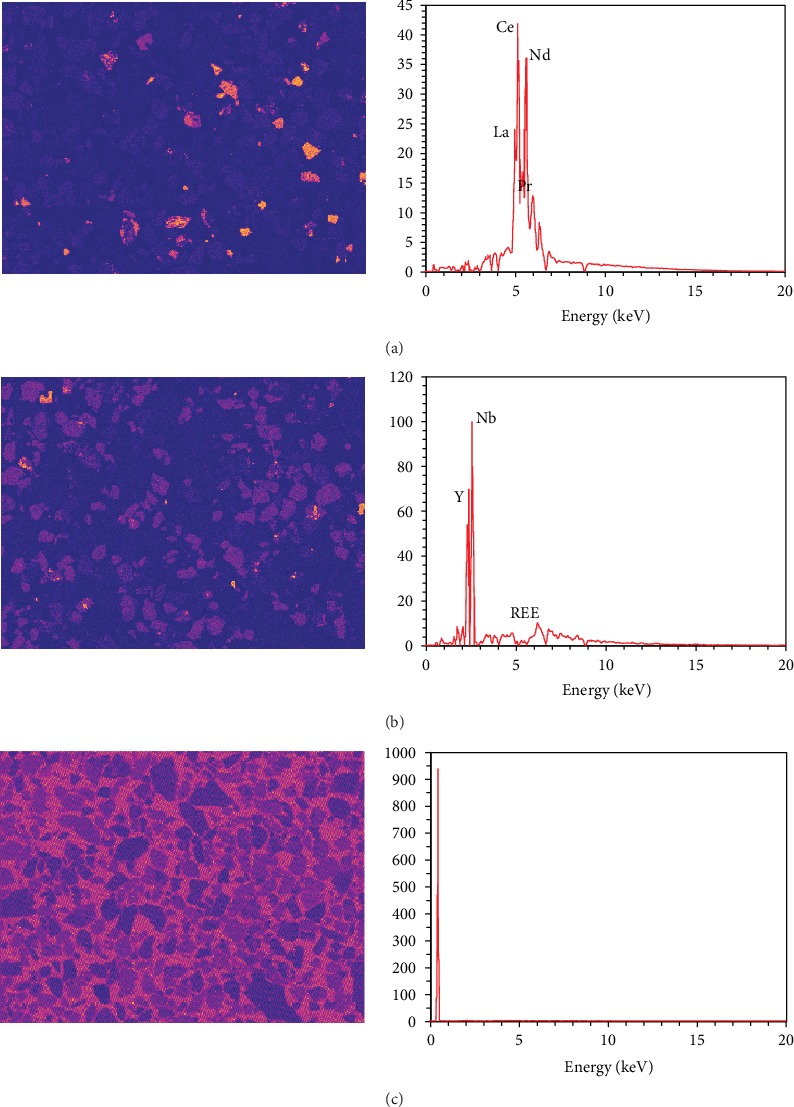
RE-components separated by NMF-based BSS: (a) bastnaesite and parisite component; (b) fergusonite component; (c) epoxy component.

**Figure 8:**
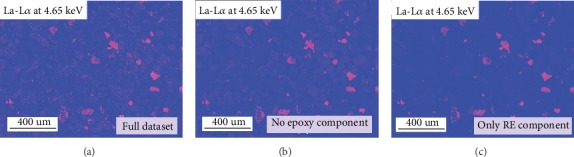
La-L*α* intensity maps retrieved from NMF datasets.

Since the ICA allows the complex cancellations between positive and negative numbers to realize the linear combination of these components, the decomposed EDS spectrum and the map may lose their intuitive physical meanings and fail to duplicate the reality [15]. By contrast, the NMF-based BSS only allows the combinations and can thoroughly avoid this issue.  Figure 7 shows the NMF RE-components, including the bastnaesite and parasite component (Figure 7(a)), the fergusonite component (Figure 7(b)), and the epoxy component (Figure 7(c)).  Figure 8 illustrates the comparison of the La-L*α* intensity maps, including the one retrieved from the full dataset (Figure 8(a)), the modified one retrieved from the dataset excluding the NMF epoxy component (Figure 8(b)), and the map retrieved from the NMF RE-components (Figure 8(c)).

**Figure 9:**
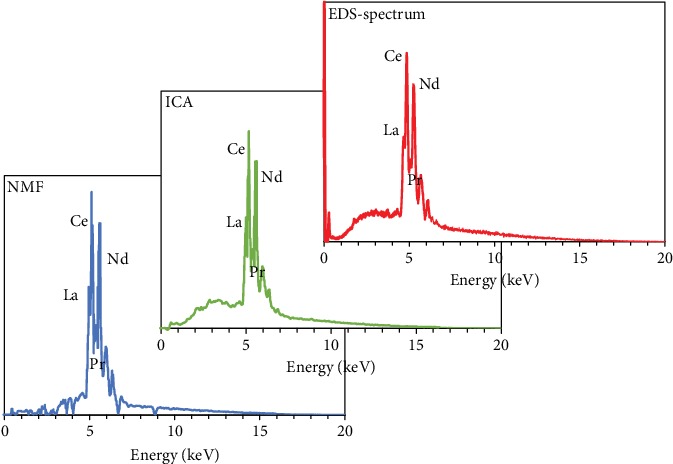
Comparison between ICA and NMF spectra and a real EDS spectrum.

As shown in Figure 7, the NMF RE-components fit better with the fact without the dark particle profiles compared with the ICA components. Regarding the X-ray intensity map, the La-L*α* map retrieved from the RE-components (Figure 8(c)) successfully gets rid of the noise from the epoxy background and the interferences from other phases. However, the NMF spectra are far from a real EDS spectrum with some sharp jumps, and by contrast, the ICA spectra have much smoother outlines. A more direct comparison is illustrated in Figure 9, which displays the ICA and NMF spectra of the bastnaesite and parisite component and a real EDS spectrum extracted from a bastnaesite phase. The spectra after the BSS treatments represent the X-ray signals of the separated components, so strange jumps are observed compared to the conventional EDS spectrum.

**Figure 10:**
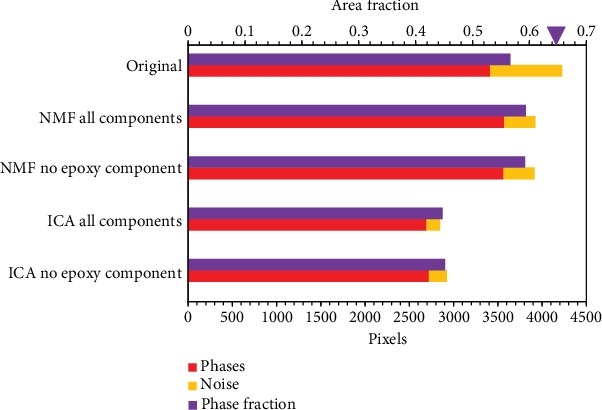
Rare earth phases (red bars) and noise (yellow bars) identified in the 5-minute map datasets. The purple bars represent the proportions of all phases identified in the 5-minute maps, and the purple triangle indicates that in the 60-minute map.

With the modified X-ray maps retrieved from the reconstructed dataset, the phases were further identified. The phase maps were processed with the different datasets reconstructed with the selected ICA and NMF components, and they are statistically compared in terms of the phase identification of the rare earth phases (red bars) and the noise (yellow bars) and the sum area fraction of all mineral phases (purple bars) in Figure 10. Two datasets were reconstructed for the ICA and NMF algorithms, respectively: one with all the components and one excluding the epoxy component. As shown in this figure, both the NMF and ICA algorithms significantly denoised the map, but there are less rare earth phases identified after the ICA treatment. Additionally, the subtractions of the epoxy background do not further improve the maps. Since the BSS calculation is aimed at recovering the sources using the observed data, the dataset separation can underscore the data structure. Thus, even without component exclusion, the mineral phases can be more outstanding just through the data decomposition and reconstruction.

Besides the identification of REMs, the proportion of all identified mineral phases (purple bars) is also an important criterion for evaluating the applied algorithms. As shown in Figure 10, the NMF algorithm can also increase the proportion of all phases, improving the identification power for the 5-minute map. The ultimate objective of this study is to find the optimal procedure to achieve an efficient phase analysis, which can render the 5-minute map with the 60-minute quality. Compared with the original 5-minte map, the BSS modified maps have great improvements and can achieve a similar quality of the 60-minute one.

## 4. Conclusion

The PCA and BSS analyses were performed on an EDS map dataset which was acquired on a REM sample for only 5 minutes. Through the denoising analysis with the PCA, a much clearer phase map was obtained. The number of remaining components should be carefully checked in order to avoid the phase omissions and save the computation time. The BSS treatments can provide a similar denoising result, but they have different principles: the PCA denoising is achieved by remaining the components with higher variances and discarding the rest as noise, but the BSS-denoising is achieved by directly excluding the undesired components.

Through the performance of BSS, the mineral phases with similar compositions were directly separated, bringing convenience to the phase identification. However, due to the complex nature of this mineral sample, an accurate component separation is not easily achieved just using the BSS. In order to avoid repetitive attempts on the data separation, it is preferred to combine the BSS analysis with the conventional phase map analysis. The components decomposed by the BSS can provide a preliminary idea of the sample, and then the phase map can be processed with the modified X-ray intensity maps which are retrieved from the reconstructed dataset. In this way, the phase identification and noise reduction can be both achieved. Through the comparisons between the ICA and NMF algorithms, the NMF is more suitable for a phase map analysis due to its smaller signal reduction.

## Figures and Tables

**Table 1:** Current condition of the map acquisitions.

Beam energy	Probe current	Collecting count rate	Total counts	
			5 min	60 min
20 kV	280 pA	67.6 kcps	20,688,661	201,809,640

## Data Availability

No data were used to support this study.
